# Drivers of Resistance in Uganda and Malawi (DRUM): a protocol for the evaluation of One-Health drivers of Extended Spectrum Beta Lactamase (ESBL) resistance in Low-Middle Income Countries (LMICs)

**DOI:** 10.12688/wellcomeopenres.17581.2

**Published:** 2023-05-02

**Authors:** Derek Cocker, Melodie Sammarro, Kondwani Chidziwisano, Nicola Elviss, Shevin T. Jacob, Henry Kajumbula, Lawrence Mugisha, David Musoke, Patrick Musicha, Adam P. Roberts, Barry Rowlingson, Andrew C. Singer, Rachel L. Byrne, Thomas Edwards, Rebecca Lester, Catherine N. Wilson, Beth Hollihead, Nicholas R. Thomson, Christopher P. Jewell, Tracy Morse, Nicholas A. Feasey

**Affiliations:** 1 Malawi-Liverpool Wellcome Trust Clinical Research Program, Blantyre, Malawi; 2 Department of Clinical Sciences, Liverpool School of Tropical Medicine, Liverpool, UK; 3 Centre for Health Informatics Computing and Statistics, Lancaster University, Lancaster, UK; 4 Centre for Water, Sanitation, Health and Appropriate Technology Development (WASHTED), Polytechnic, University of Malawi, Blantyre, Malawi; 5 Department of Civil and Environmental Engineering, University of Strathclyde, Glasgow, UK; 6 Science Group, United Kingdom Health Security Agency, London, UK; 7 Global Health Security Department, Infectious Disease Institute, Makerere University, Kampala, Uganda; 8 Department of Medical Microbiology, College of Health Sciences, Makerere University, Kampala, Uganda; 9 College of Veterinary Medicine, Animal Resources and Biosecurity (COVAB), Makerere University, Kampala, Uganda; 10 Conservation & Ecosystem Health Alliance, Kampala, Uganda; 11 Department of Disease Control and Environmental Health, College of Health Sciences, Makerere University, Kampala, Uganda; 12 Wellcome Trust Sanger Institute, Wellcome Trust Genome Campus, Hinxton, Cambridge, UK; 13 Department of Tropical Disease Biology, Liverpool School of Tropical Medicine, Liverpool, UK; 14 UK Centre for Ecology & Hydrology, Benson Lane, Wallingford, UK; 15 Centre for Drugs and Diagnostics, Liverpool School of Tropical Medicine, Liverpool, UK; 16 Faculty of Health and Life Sciences, University of Liverpool, Liverpool, UK; 17 Department of Pathogen Molecular Biology, London School of Tropical Medicine and Hygiene, London, UK

**Keywords:** Antimicrobial Resistance, Africa, One Health, Environment

## Abstract

In sub-Saharan Africa (sSA), there is high morbidity and mortality from severe bacterial infection and this is compounded by antimicrobial resistance, in particular, resistance to 3rd-generation cephalosporins. This resistance is typically mediated by extended-spectrum beta lactamases (ESBLs). To interrupt ESBL transmission it will be important to investigate how human behaviour, water, sanitation, and hygiene (WASH) practices, environmental contamination, and antibiotic usage in both urban and rural settings interact to contribute to transmission of ESBL E. coli and ESBL K. pneumoniae between humans, animals, and the environment.

Here we present the protocol for the Drivers of Resistance in Uganda and Malawi (DRUM) Consortium, in which we will collect demographic, geospatial, clinical, animal husbandry and WASH data from a total of 400 households in Uganda and Malawi. Longitudinal human, animal and environmental sampling at each household will be used to isolate ESBL E. coli and ESBL K. pneumoniae. This will be complimented by a Risks, Attitudes, Norms, Abilities and Self-Regulation (RANAS) survey and structured observations to understand the contextual and psychosocial drivers of regional WASH practices.

Bacterial isolates and plate sweeps will be further characterised using a mixture of short-,long-read and metagenomic whole-genome sequencing. These datasets will be integrated into agent-based models to describe the transmission of EBSL resistance in Uganda and Malawi and allow us to inform the design of interventions for interrupting transmission of ESBL-bacteria.

## Introduction

Antimicrobial resistance (AMR) is a huge and complex global public health problem ^1^. It is a threat to health that reflects both the interconnectedness of humans, animals and the environment and humanity’s dependence on antimicrobials ^2^. In sub-Saharan Africa (sSA), there is a high incidence of severe bacterial infection, frequently inadequate health system infrastructure to diagnose and treat bacterial disease, and widespread and uncontrolled availability of antimicrobials, which drives antibiotic use (ABU) in both human and animal sectors ^3, 4^. There is also inadequate water, sanitation and hygiene (WASH) infrastructure to mitigate spread of environmentally dependent bacteria between humans, animals, and the environment ^5^. This situation favours the transmission of AMR-bacteria, but the relative contribution of these different factors is uncertain.

The 3 ^rd^-generation cephalosporin (3GC) ceftriaxone is frequently the antimicrobial agent of choice for the empirical management of sepsis across much of sSA ^6, 7^. 3GC resistant (3GC-R) enteric bacteria have rapidly emerged, largely due to acquisition of genes encoding extended-spectrum beta lactamase (ESBL)-producing enzymes, resulting in infections that are frequently locally untreatable, due to unavailability of carbapenems or other reserve antibiotics ^8^. ESBL-producing *Escherichia coli* and *Klebsiella pneumoniae* are key examples of this. As low-income countries (LIC) in Africa have poor access to watch and reserve agents ^7, 9^, it is critical to define the relative importance of different transmission routes of ESBL-producing enteric bacteria in order to develop interventions that will interrupt pathogen transmission and ultimately prevent drug resistant infections (DRI).

Uganda and Malawi are LIC with a high burden of disease from sepsis and malaria, high prevalence of HIV, poorly regulated antimicrobial markets, and inadequate WASH infrastructure ^5, 10– 14^. Here, we present the protocol developed by the Drivers of Resistance in Uganda and Malawi (DRUM) Consortium. DRUM will work in urban, peri-urban, and rural settings in Uganda and Malawi and focus on the isolation and genomic characterisation of ESBL producing *E. coli* (ESBL-E) and *K. pneumoniae* (ESBL-K). These bacteria were selected as they belong to the same family and often share AMR phenotypes, however *E. coli* is typically considered to be both community-acquired and nosocomial, whereas *K. pneumoniae* is more often judged to be the archetypal nosocomial AMR pathogen ^15^.

We will take an interdisciplinary, One-Health approach to assess how human behaviour, WASH practices, environmental contamination with ESBL bacteria, and ABU in urban and rural locations within Uganda and Malawi contribute to the transmission of ESBL-E and ESBL-K between humans, animals, and the environment and how this transmission relates to strains isolated from the blood of humans with drug-resistant infection (DRI). We will collect demographic, geospatial, WASH, longitudinal clinical and molecular microbiological data, and integrate these data into agent-based models designed to estimate the impact of putative interventions on interrupting transmission of ESBL bacteria.

### Aim

**Figure 1.**
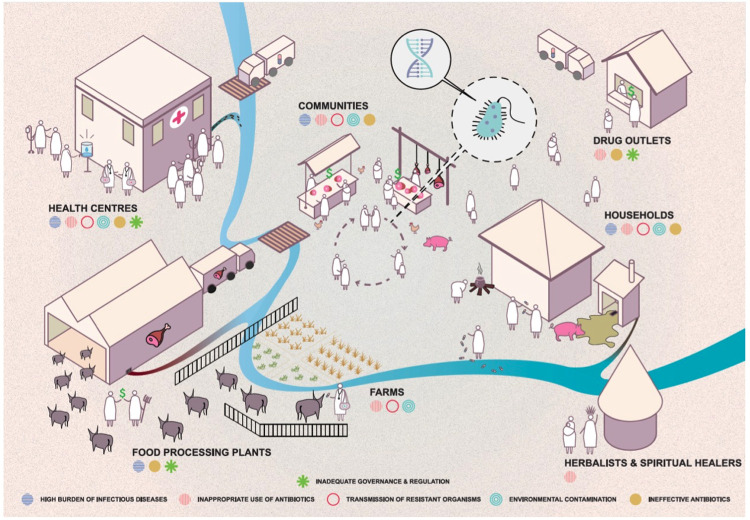
Hypothetical model of related behaviours and the movement of AMR-bacteria in Uganda and Malawi. The schematic situates the household at the heart of the model, in which humans act in response to their environment within which bacteria are evolving in response to selective pressures around them.

In order to determine the critical points at which efforts to interrupt human AMR acquisition are likely to have the greatest impact in Eastern Africa and beyond, we hypothesise that the household is a key setting in which ESBL enteric bacteria are transmitted. We therefore aim to identify risk factors for and infer drivers of ESBL-E and ESBL-K transmission in Uganda and Malawi at the household level. This is summarised in Figure 1, created following a stakeholder meeting in Uganda in 2018 by Design Without Borders.

### Site selection

**Figure 2.**
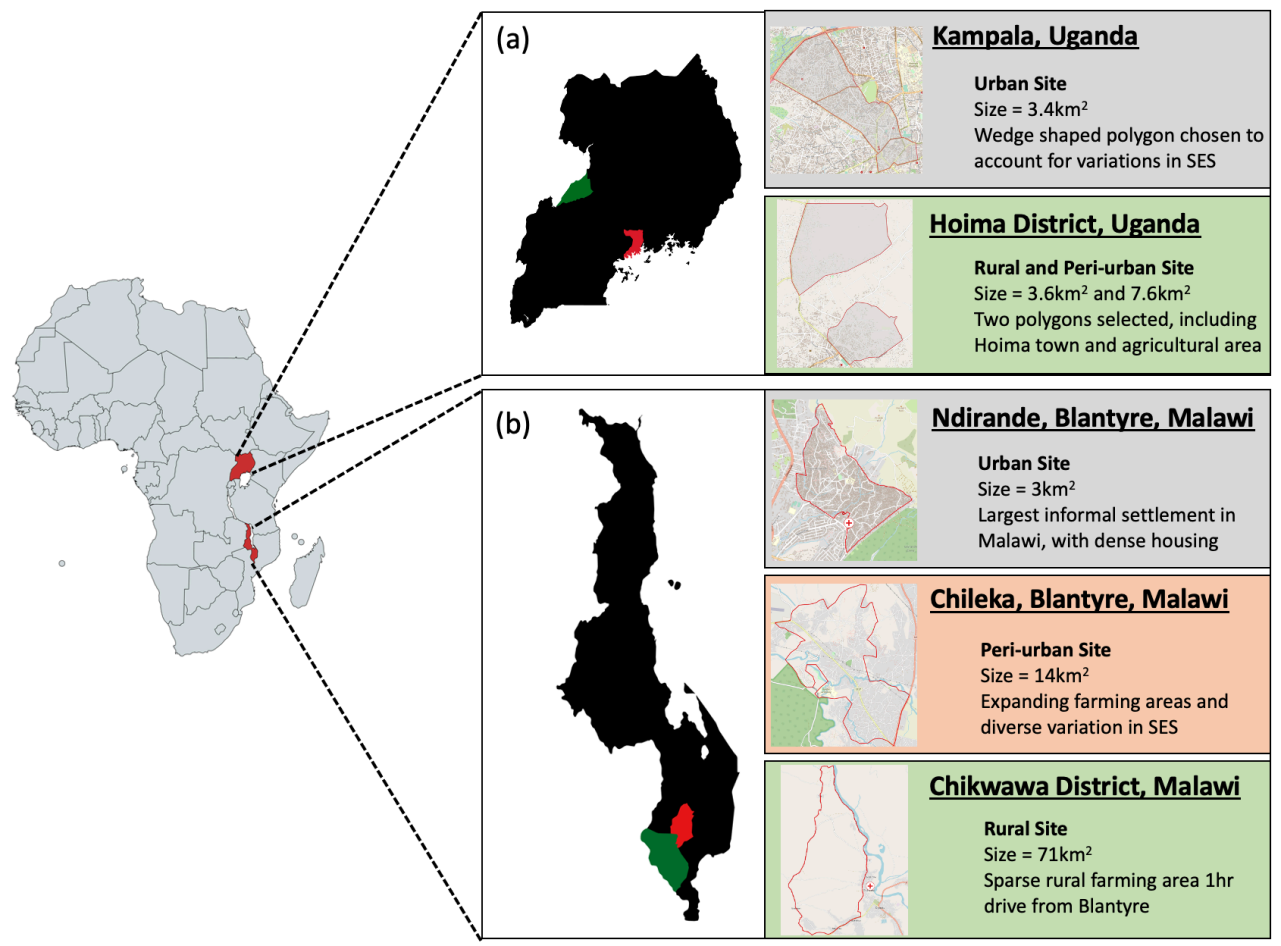
Diagram of DRUM study sites. ( **a**) We selected two geographic areas within Uganda including Kampala (red) and Hoima District (green). From these areas, polygons were created that mapped an urban setting (Kampala) and peri-urban/rural setting (Hoima District). ( **b**) We selected sites in two regions within southern Malawi including Blantyre (red) and Chikwawa District (green). Polygons were created and mapped for urban (Ndirande) and peri-urban (Chileka) settings within Blantyre and a rural setting within Chikwawa District.

DRUM consortium members identified sites representing urban, peri-urban, and rural settings to enable variations in WASH behaviours, animal husbandry practices, ABU, and household and broader environmental contamination with ESBL-producing bacteria to be contrasted. Additionally, sites were considered based on perceived acceptability of research within the communities and existing research capacity. Therefore, in Malawi, Ndirande (urban) and Chikwawa (rural) were selected because of the opportunity to utilize data from previous studies (i.e. detailed censuses) and prior research engagement, and Chileka (peri-urban) was selected due to local prior knowledge. We sought to achieve a comparable mixture in Uganda with varied socioeconomic status in Kampala (urban) and Hoima District (peri-urban and rural). Within these sites, recruitment polygons were drawn from local administrative wards ( Figure 2).

### Malawian site descriptions

Healthcare is free at the point of delivery in Uganda and Malawi, and this should be assumed unless otherwise stated.

***Site 1: Ndirande, Blantyre, Malawi (Urban)***

Ndirande is a large urban settlement with high-density housing 4 km from the geographical centre of Blantyre, the second largest city of Malawi ^16, 17^ and where 15% (109,164) of the Blantyre population resides ^18^. Healthcare is provided by one large, government Health Centre (Ndirande Health Centre) and by the tertiary referral hospital for the Southern region, Queen Elizabeth Central Hospital (QECH), 2–6 km away ^16, 19^. HIV prevalence in adults aged 15–65 is 18% and there is a high burden of typhoid and tuberculosis ^20, 21^. According to the Malawian Health Management Information System report, malaria accounts for about 34% of all outpatient visits and 18% of all hospital deaths in Malawi ^22^. The study polygon is 3 km ^2^, and our initial survey in April-May 2019 identified 8 secondary schools, 46 primary (or nursery) schools, 52 places of worship, 15 markets, 1 farm and 9 pharmacies within it.

***Site 2: Chileka, Blantyre, Malawi (Peri-Urban)***

Chileka is a peri-urban administrative ward on the northern outskirts of Blantyre city. Healthcare is provided by a government Health Centre (Chileka Health Centre), a small local private hospital (Mtengo-Umodzi) or admission to QECH 10–16 km away. The study polygon is 14 km ^2^, and our initial survey in April-May 2019 identified 3 secondary schools, 20 primary (or nursery) schools, 14 places of worship, 4 large farms and 6 pharmacies within it.

***Site 3: Chikwawa, Malawi (Rural)***

Chikwawa is a large district with a population of ~450,000, situated in the southern Shire valley and its border is 50 km from Blantyre ^23^. It is a rural area, including a mixture of subsistence and large-scale sugar farming, and given its low-lying situation is historically prone to flooding ^24^. Healthcare is provided by Chikwawa District hospital, 14 health centres and 26 community health care worker outposts ^23^. We identified a 71 km ^2^ study polygon readily accessible from Blantyre by road, including villages engaged in research activity on the edge of Chikwawa town. Our survey in April-May 2019 identified 2 secondary schools, 9 primary (or nursery) schools, 29 places of worship, 3 markets, 11 farms and 1 pharmacy within the polygon.

### Ugandan site descriptions

***Site 4: Kampala, Uganda (Urban)***

Kampala, the capital and largest city of Uganda has a metropolitan area population of 3.3 million people. Adult HIV prevalence is 6.9% ^25^. In 2020, Uganda had the 3rd highest global burden of malaria cases and deaths (5.4%) and the 5th highest level of deaths (3.5%) ^12^. The sampling frame comprises of 3 contiguous areas drawn in wedge shape (measuring 3.4 km ^2^ x 2.7 km ^2^ x 1 km ^2^) with a spectrum of population density areas. These areas were loosely stratified relative to each other as being of low, medium or high socioeconomic status based on local knowledge. The smallest polygon closest to the centre is considered low, whilst the one furthest from the centre as medium and the middle one as high socioeconomic status.

***Site 5: Hoima, Uganda (Rural and Peri-urban)***

Hoima, in the Western Region of Uganda, is the main municipal, administrative, and commercial centre of Hoima District and has a population of 122,700 people ^26^. HIV prevalence among adults aged 15–64yrs in the Mid-West Region of Uganda where Hoima is located is 5.7% ^25^. The sampling frame comprises of two non-contiguous polygons of 3.6 km ^2^ and 7.6 km ^2^, the former incorporating Hoima town (peri-urban) and the latter (rural) being a few kilometres away from Hoima town and which has more animal and human cohabitation.

## Methods

### Household selection process

As DRUM will investigate AMR transmission at the household level, we chose a spatial design based on the “inhibitory with close pairs” approach ^27^. This enables us to distribute primary sampling sites across the study area evenly, avoiding systematic biases that may occur when sampling on a regular grid. Secondly, “close-pair” points are added to the design to allow localised comparison of sample sites and therefore measurement of close-range correlation in AMR status. Thus, seventy percent of households will be sampled at a minimum inhibitory distance (MID) from all other points ^28^. Using one inhibitory point at a time, the rest of the points, called close pairs, are randomly selected within a circle with a pre-determined close-pairs radius (CPR). The minimum distance for our design is 100 meters and the radius for each close pair is 30 meters. These values were chosen based on results from a spatial investigation of enteric pathogen *Salmonella* Typhi in Blantyre that showed a spatial correlation up to approximately 150 meters ^29^.

Depending on the richness of existing geospatial data within each study area, we will implement different versions of the algorithm in each area. In Ndirande (Malawi), where all households had previously been geolocated, direct random sampling of households subject to the spatial constraints above is possible ^16^. In Hoima (Uganda), where OpenStreetMap (OSM) data appears complete, OSM-derived building locations can be chosen to identify potential households. In Chikwawa (Malawi), WorldPop population density rasters allow us to preferentially (though not exclusively) propose sampling sites in high population density areas thus avoiding field teams visiting vacant sites ( www.worldpop.org/). In Kampala (Uganda) and Chileka (Malawi), apparent uniformity of the population density across the study area allows a simple spatially uniform proposal to be used. Two practical site-specific considerations are necessary. Firstly, for Chileka, the MID and CPR must be doubled due to the sparse population. In Kampala, the availability of a marked socioeconomic gradient within the study region allows stratification of the population by socioeconomic status, with households randomised within strata, but respecting our spatial design constraints across strata borders.

Proposed sampling locations are then translated into households by the data collection field teams. For instances where either no suitable household exists at the location or in the event that a household declines to participate in the study, a random direction is selected by the field team, and the closest consenting household in that direction is chosen.

### Recruitment of households

**Figure 3.**
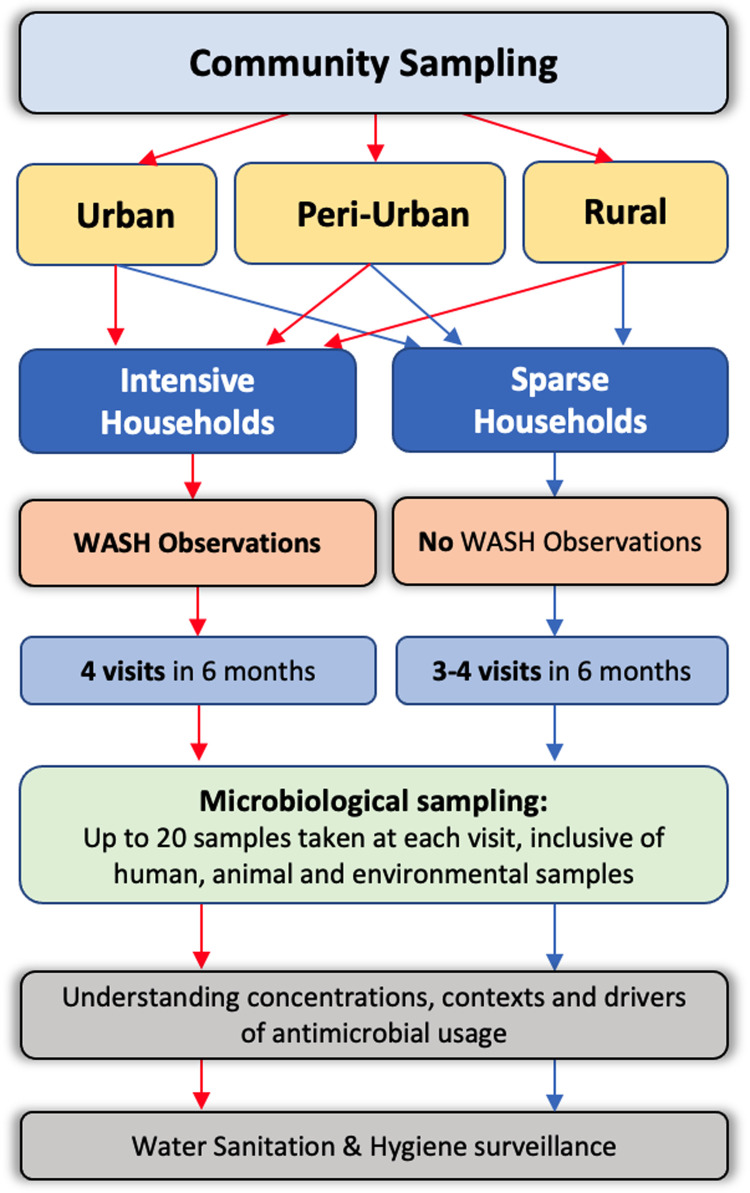
DRUM household study design.

We aim to enrol up to 100 households in each of the five sites. Households will be grouped into either “intensive” or “sparse”, with 15 intensive households pre-selected at random within each polygon, and all others allocated as sparse ( Figure 3). Intensive households will undergo extensive WASH observations at the first and last visit, whereas “sparse” households will not undergo any WASH observations ( Figure 3).

All households will be followed up at 3–4 time points over a period up to 6 months to provide longitudinal microbiological and WASH data. Household recruitment will be staggered over 12 months to assess seasonality of transmission of ESBL-bacteria. At each visit, households will be asked to respond to questionnaires to provide information at the individual and household level on ABU, health seeking behaviour and WASH behavioural practices. Microbiological sampling will be undertaken to determine the presence of ESBL *E. coli* and ESBL *K. pneumoniae* from human, animal and environmental samples.

### Participant eligibility

Eligibility will be considered at the level of the household and individuals. Households will be required to exist within the boundaries of the study polygon and be able to provide a minimum of 12 samples at the baseline visit, inclusive of a minimum of 2 human stool samples from household members. Individuals will be required to speak either the predominant local language (Chichewa in Malawi or Luganda or Runyoro in Uganda) or English to provide informed consent, and not have confirmed or suspected acute bacterial infection at the time of recruitment.

### Data collection

***Case Report Forms (CRFs)***

Study CRFs have been designed by an interdisciplinary working group of the DRUM consortium that included specialists in human health, animal health, food, water and environmental microbiology, WASH & Environmental health and medical anthropology. Questions were selected from pre-tested tools evaluating regional demographics, human and animal health, WASH infrastructure and behavioural practices, humans and animal ABU determinants and environmental exposures ^29, 30^. These questions were inputted into CRFs that were tailored to the resident population, structured into either individual or household level, thematically separated into key drivers of AMR and translated into local languages ( Table 1).

**Table 1.** DRUM CRF themes and data capture.

	Individual Level Data	Household Level Data
**Demographic**	· Participant Demographics	· Household Demographics · Socio-Economic Information · Household Head Information
**Health**	· Health Status and Comorbidities · Regular Medication Use · Recent Illness	· Household Health Seeking Behaviour
**Exposure Risk**	· Healthcare Exposure · Travel and Residency · Health Seeking Behaviour	· Visitors into the household
**Antibiotic Usage**	· Antibiotic Usage	· Household Experience of Illness and Antibiotics
**WASH**	· Hand-Washing Data	· Household WASH Infrastructure · Toileting Behaviour · Waste Management · Water Usage and Management · Washing and Bathing Practices · Food Preparation and Hygiene Information. · Hand-Washing Data
**Environmental**		· Household Infrastructure · Household Environment
**Animal**		· Household Animal Husbandry · Animal Health and Disease Prevention · Drug Usage in Household Animals (including antibiotics)

At the baseline visit, these CRFs will be completed to provide information at the individual and household level on human health, ABU, socioeconomic status, health seeking behaviour, structural and behavioural WASH practices and animal husbandry (Extended data). At each follow-up visit, changes to human health, household practices and antibiotic exposure will be assessed (Extended data).

***Longitudinal microbiological sampling***

**Figure 4.**
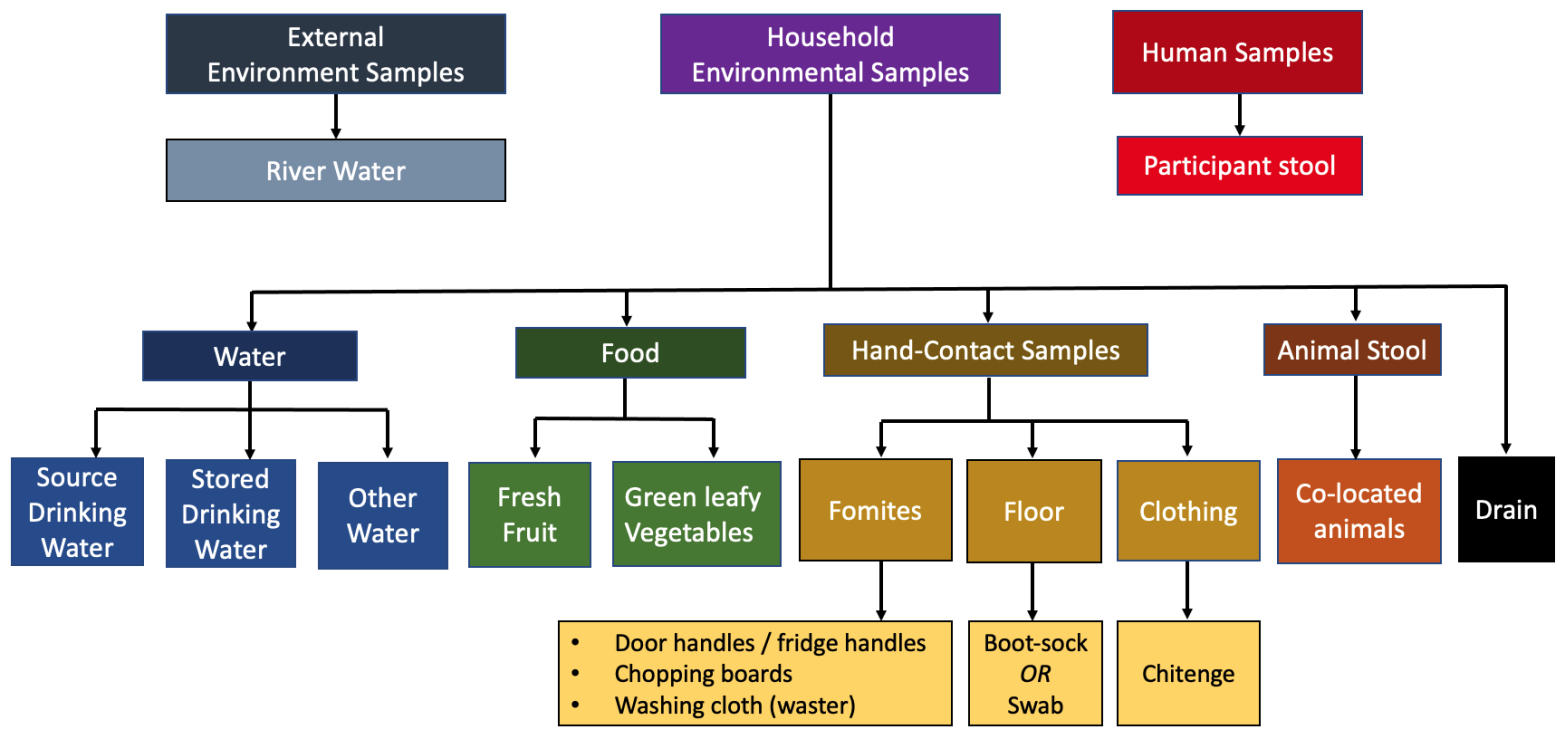
DRUM microbiological sampling frame, used at household visits. Samples are inclusive of human stool, animal stool, and a selection of household environment and the broader external environment.

The consortium was asked to consider priorities for household sampling at the kick-off meeting at Liverpool School of Tropical Medicine (LSTM), UK (23/09/2018). We decided to focus on areas identified as hand-contact zones or where food handling occurred and also to include broader environmental sites that we hypothesise serve as important reservoirs of ESBL-producing organisms. We established a consensus opinion for the microbiological sampling strategy based on a maximum of 20 samples per visit, inclusive of human and animal stool samples and environmental samples (see Figure 4).

**Field sampling methods**

Human stool will be self-collected in a 30 mL stool pot by participants. Animal stool samples will either be collected using rectal/ cloacal swabs (for poultry) or taken directly from the ground. Food samples will be placed in sterile Whirl-Pak® bags, and all water samples will be collected using sterile 500 ml Nalgene® BPA-free, polypropylene bottles.

Household environmental sampling will be informed by the WASH observations to determine high-risk areas of environmental contamination. Environmental contact-surface samples and clothing samples will be collected with 3M™ Sponge-Sticks containing 10 ml sterile buffered peptone water (BPW) broth, and floor samples will be collected with the use of boot socks. Outer drain samples (defined as water in motion either in a constructed drain (dug or built) or moving on a surface) will be collected in a 30 ml universal container from within the household compound. Detailed descriptions of the sampling processes are included in the study Standard Operating Procedures (SOPs) in the Extended material.

All samples will be issued a unique identification code, labelled, stored in ice chests at 2–8 ^o^C in the dark and transported to the laboratory, for processing within 24 hours, where possible.

**Microbiological Methodology**

Human stool, animal stool and environmental swabs will not require pre-processing steps. BPW (Oxiod) will be directly added to the sample upon reception. Water and food samples will be pre-processed as follows:

- Water samples will be filtered through a sterile 0.45 µm cellulose-ester gridded membrane (VWR™) using a vacuum-based manifold, before adding to universal containers with 20 ml of BPW (Oxiod). In river water samples, a second sample will be processed in parallel, and the filter membrane will be stored at -80°C without the addition of BPW.
- Fruit (whole) will have enough BPW (Oxiod) added to the Whirl-Pak® bag to cover before being manually stomached for a period of 30 sec to 3 min. The fruit will then be aseptically removed from the bag prior to incubation.
- Green leafy vegetables will be weighed and have nine times the weight of the food added in BPW (Oxiod) to obtain a sample-to-diluent weight ratio of 1:9, before being manually stomached for 30 sec to 3 min. Vegetables will be left inside the Whirl-Pac® bag while incubated.

Consistent with practice at the UK Health Security Agency Food Water and Environment (FWE) Microbiology Services, all samples will initially be cultured in enrichment broth (BPW) to improve the recovery of Gram-negative organisms. The volumes of BPW (Oxiod) added will depend on sample type and will be determined by either the manufacturer’s advice (3M™ Swab-Samplers), expert opinion and SOPs from FWE (3M™ Sponge-Sticks, water filtration methods, food processing methods), previous local experience (stool processing) or from pre-testing and optimisation in the piloting phase of the study (river water processing, drain sample processing, boot socks) (see Extended material).

Once the enrichment broth (BPW) has been added, all samples will be placed in an aerobic incubator at 37 ± 1 ^o^C for 18–24 hours. After incubation a 1.8 ml aliquot of the culture BPW will be stored at -80°C, and a 1μl loop of the remaining sample will be plated onto ESBL CHROMagar™ chromogenic agar (CHROMagar™, France). Plates will be placed in an aerobic incubator at 37 ± 1 ^o^C for 18–24 hrs and read for growth of ESBL bacteria, via the presence of either pink, blue or white colonies. Pink colonies and (indole positive) white colonies will be categorised as ESBL *E. coli* while blue colonies will undergo speciation for *K. pneumoniae*, using high resolution melt-curve (HRM) PCR (QIAGEN, USA), to identify ESBL *K. pneumoniae* isolates ^31^. ESBL status will be inferred by chromogenic methods alone and not formally confirmed by phenotype, however isolates growing on ESBL media will be whole genome sequenced. A plate sweep will be taken from each ESBL CHROMagar™ plate that has positive growth of ESBL colonies. ESBL isolates and plate sweeps will be stored at -80°C.

Samples will be stored at intervals during the microbiological processing to facilitate subsequent whole genome sequencing (WGS), including aliquots of the original sample (shotgun metagenomics); samples pre-enriched with BPW (limited-diversity metagenomics via mSWEEP/mGEMS), CHROMagar™ plate sweeps (limited-diversity metagenomics) and single colony picks (short-read and long-read sequencing) ^32^.

**DNA extraction, Library preparation and Sequencing**

DNA will be extracted from all ESBL-positive isolates, plate sweeps and pre-enriched BPW ESBL positive samples using the QIASymphony DSP Virus/Pathogen mini-kit® on the QIASymphony® (QIAGEN, USA) automated DNA extraction platform or manually extracted using the DNeasy® blood and tissue kit (QIAGEN, USA). Extracted DNA will be shipped to the Wellcome Sanger Institute (WSI, UK) under export licences issued following signature of Access and Benefit Sharing agreements in accordance with the Nagoya protocol. 

All DNA samples will be quantified with Biotium Accuclear® Ultra high sensitivity dsDNA quantitative kit (Bioitium, USA) using Mosquito LV liquid platform (SPT LabTech, UK), Agilent Bravo WS (Agilent Technologies, USA) and BMG FLUOstar Omega plate reader (BMG LabTech, Germany) and cherrypicked to 200ng / 120ul using Tecan liquid handling platform (Tecan, Switzerland). Cherrypicked plates will be sheared to 450bp using a Covaris LE220 (Covaris, USA) instrument and samples purified using Agencourt AMPure XP SPRI beads Beckman Coulter, Inc., USA) on Agilent Bravo WS. Libraries will be constructed using the NEB Ultra II custom kit (New England Biolabs, USA)

DNA from single colony pick isolates will be whole genome sequenced on the Illumina X10 platform (Illumina Inc, California, USA) to produce 150bp paired end short reads. Preliminary analysis of these short-read WGS data will inform the identification of clusters from which representative isolates will be selected for long read sequencing on the MinION platform (Oxford Nanopore Technologies, UK) in order to generate hybrid, improved draft assemblies, and thus characterise mobile genetic elements (MGEs). Finally, shotgun metagenomic and limited diversity metagenomic (plate sweeps) sequencing will be performed on up to 420 pre-enriched BPW samples and 1500 ESBL selected plate sweep samples on the Illumina HiSeq 4000 platform (Illumina Inc, California, USA) to investigate within host microbial diversity and AMR gene pool or “resistome”.

***WASH Evaluations***

**Household WASH, environmental health and food safety evaluations**

Each recruited household will be asked to engage with a range of qualitative and quantitative data collection methods to gain an understanding of the contextual and psychosocial elements of their household, individual and habitual WASH practices as outlined in IBM-WASH ^33^. Questions will be asked of household members at the baseline assessment (combined with the household and individual CRFs), and a checklist and sanitation inspection form will be completed by a member of the study team at each visit to evaluate WASH infrastructure. Lastly, a household plan will be completed at baseline to contextualise the household infrastructure where specific activities take place (including perceived high-risk areas) and aid in analysis.

WASH practices will be assessed via checklist and structured observations at households and identified for “intensive surveillance”, at both the baseline and fourth visit. Observations will be undertaken on 3 consecutive days, for a period of 6 hours per day, with two morning sessions (6am-12pm) and one afternoon session (12pm-6pm) to describe WASH practice over the period of a day. The focus on early sessions has been chosen due to previous studies illustrating that key WASH activities occurred mainly in the mornings ^34^. Observations will be recorded by research staff and summarised in a structured format for content analysis to enable the identification of critical control points around WASH behaviours for faecal and environmental exposure.

**Understanding WASH behavioural drivers**

Psychosocial drivers of WASH practices will be explored using the Risks, Attitudes, Norms, Abilities and Self-Regulation (RANAS) Model, undertaken at up to 100 households in each region ^35, 36^. The RANAS questionnaire design will be informed by the structured observations in intensive households and focus on hand hygiene, food preparation, waste management, water usage and environmental exposure. The RANAS survey will be conducted with 2 household members in each household, and where possible, will be directed to the household head (e.g. father) and one household worker (household staff member). RANAS data will be analysed using an ANOVA mean comparison to determine the differences between doer and non-doer contextual and psychosocial factors for potential targeted behaviours. The data from this survey will be used to inform potential behaviour change techniques which could be used to tackle high risk transmission areas identified in the agent-based model.

***Assessment of broader environmental exposure***

Transect walks of each region will be undertaken using an integrated approach to the collection and evaluation of environmental, WASH and microbiological data to understand the wider context in which household members are living. Based on the principles of the SaniPath method, walks will be undertaken with community leaders using walking interviews, while collecting video footage and photographs and geolocating walk routes and sampling sites. Reference will be made to specific Shit Flow Diagrams (SFD), where available, which visually describe excreta flow in urban and rural settings, and data will be mapped to provide a spatial outline of potential pathways for faecal exposure ^37– 39^. Wherever feasible, longitudinal data will be collected on study sites to assess the effects of seasonality. This novel adaption of the SaniPath tool will enable us to integrate environmental AMR data into urban and rural WASH exposure pathways.

Observations and structured checklists will be completed at 10 public and institutional settings within each of the five sites (n=50). This will be complemented by Focus Group Discussions (FGDs) and In-depth interviews (IDIs) with key informants (heads of household, primary caregivers, school children, market vendors, etc.) to explore perceptions of barriers and challenges to WASH posed by circumstances of daily life.

***Spatial analysis and integration of datasets into an agent-based model***

The initial approach will be to determine variables that have strong associations with ESBL status using model-based statistical analysis ^40^. Spatial and temporal correlations will be accounted for using both geostatistical and agent-based modelling techniques to increase the precision of our inference, and hence insight into the main demographic and environmental drivers of transmission and carriage. Geostatistical models will initially be used as a phenomenological way of detecting such associations in the quantitative elements of our data ^41^. Findings from our qualitative components will then be used to inform the structure of an agent-based model. This will allow us to test different systems models of social and behavioural features of the population that may contribute to ESBL emergence, transmission, and colonisation/decolonisation of individuals ^41^.

### Data management and analysis

**Figure 5.**
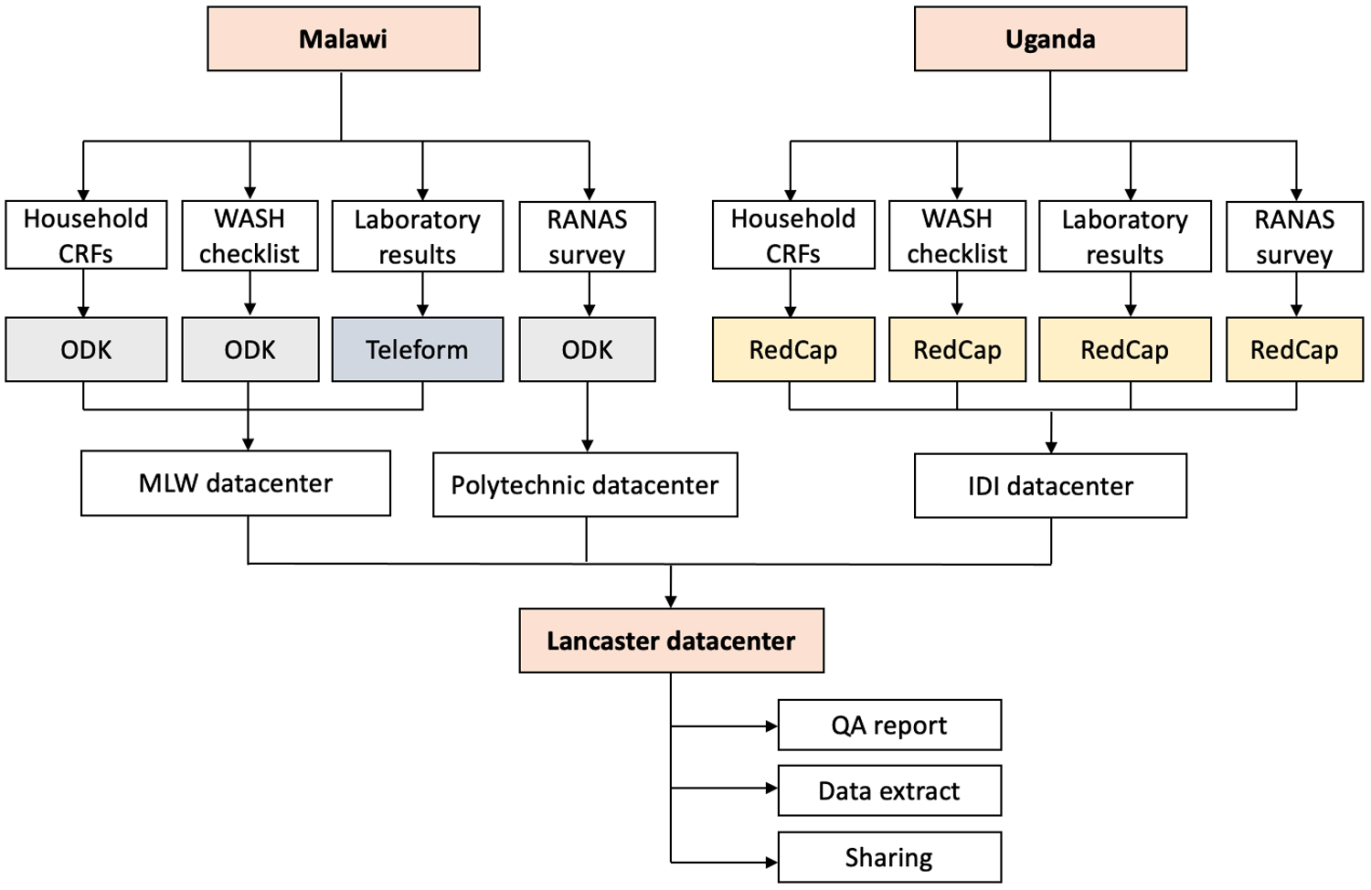
DRUM data management workflow.

In Uganda CRF data and laboratory data will be collected using REDCap (version 10.0.25). In Malawi CRF data will be collected using tablets with Open Data Kit software (ODK, 1.4.10) and laboratory data will be collected using Teleform Data Capture software (10.7). Initial transcription (where needed) and data cleaning will be performed within the local data centres in Uganda and Malawi, close to the data collection context. These data will then be pulled nightly from the local data centres to the University of Lancaster (UoL), UK and formalised into an SQL database to facilitate full record linking with RANAS and WASH study data, extract query construction, and final quality assurance ( Figure 5). All data will be securely stored with restricted access to the study PIs and database administrators at Malawi-Liverpool Wellcome Trust (MLW, Malawi), IDI (Infectious Diseases Institute, Uganda) and Lancaster, and shared where required with specific members of the DRUM project team using a secured instance of Dataverse hosted on UoL servers.

### Community engagement and involvement

Prior to study initiation and at regular intervals throughout the study, programme-wide community engagement and involvement will be held at study sites in Uganda and Malawi, including the convening of community advisory groups and meetings with the local leadership, district health offices and district executive councils. Findings will be shared with participants, communities and local government, including key stakeholders such as the Malawian Ministry of Health AMR technical working group, the University of Malawi and the Uganda National One Health Platform’s national AMR Sub-committee of the One Health Technical Working Group within Makerere University College of Health Sciences (CHS) and College of Veterinary, Animal resources and Biosecurity (COVAB).

### Ethics statement, regulatory approvals and governance

The protocol, participant information sheets, consent forms and data collection tools have been approved by the LSTM Research and Ethics Committee (REC, #18-090), College of Medicine REC, Malawi (#P.11/18/2541), Institutional Animal Care and Use Committee (IACUC), Uganda (Ref: SVARREC/18/2018), Joint Clinical Research Centre (JCRC) REC, Uganda (#JC3818) and Uganda National Council for Science and Technology (UNCST, #HS489ES).

In addition, administrative permissions have been granted from community leaders and support obtained from local community advisory groups. Sensitizations of study areas will be conducted prior to initiation and full informed written consent will be obtained from all participants recruited into the study, in their local language when required.

## Study status

In Uganda and Malawi, household recruitment and follow-ups have been completed in line with this protocol. Observational, CRF and microbiological data have been collected, cleaned, and integrated into the SQL database. RANAS questionaries and transect walks have been undertaken, and genomic and spatial analysis is underway. The available data has been fed into agent-based models, which are underdoing iterative developments and optimisation.

## Conclusion

In settings where there is a high incidence of severe bacterial infection and inadequate WASH infrastructure, we will identify risk factors and infer drivers of ESBL-E and ESBL-K transmission in Uganda and Malawi at the household level.

This one-health study will also provide insights on how human behaviour, WASH practices, environmental contamination, and ABU in urban and rural locations within Malawi and Uganda contribute to the transmission of ESBL-E and ESBL-K between humans, animals, and the environment. By integrating this high-quality data into agent-based transmission models, we will be able to determine critical points at which efforts to interrupt human ESBL acquisition are likely to have the greatest impact in sSA and share this information with policymakers to co-produce future intervention strategies.

## Data Availability

No data are associated with this article. Zenodo: Case report forms (CRFs) used for the publication: Drivers of Resistance in Uganda and Malawi (DRUM): A protocol for the evaluation of One-Health drivers of Extended Spectrum Beta Lactamase (ESBL) resistance in Low-Middle Income Countries (LMICs), https://doi.org/10.5281/zenodo.7836247 ^42^. This project contains the following extended data: DRUM01 Participant Enrolement CRF V2.pdf DRUM02 Household Enrolement CRF V2.pdf DRUM03 Household WASH CRF V2.pdf DRUM04 Participant Follow-up CRF.pdf DRUM05 Household Follow-up CRF.pdf DRUM06 Human Stool Sample Collection CRF.pdf DRUM07 Animal Stool Sample Collection CRF.pdf DRUM08 Household Food Sample Collection CRF.pdf DRUM09 Household Environmental Sample Collection CRF.pdf DRUM10 Household Floor Sample Collection CRF.pdf DRUM11 Household Clothing Sample Collection CRF.pdf DRUM12 Household Water Sample Collection CRF.pdf DRUM13 River Water Sample Collection CRF.pdf DRUM14 Household Hand-Hygiene Audit CRF.pdf Zenodo: Laboratory standard operating procedures (SOPs) used for the publication: Drivers of Resistance in Uganda and Malawi (DRUM): A protocol for the evaluation of One-Health drivers of Extended Spectrum Beta Lactamase (ESBL) resistance in Low-Middle Income Countries (LMICs), https://doi.org/10.5281/zenodo.7561679 ^43^. This project contains the following extended data: DRUM_Collection_SOP1 V2 Household human stool sampling.pdf DRUM_Collection_SOP2 V2 Household animal stool sampling.pdf DRUM_Collection_SOP3 V2 Household environmental sampling.pdf DRUM_Collection_SOP4 V2 River water sampling.pdf DRUM_SOP1_V2 Human and animal stool processing.pdf DRUM_SOP2_V2 Environmental sample processing .pdf DRUM_SOP3_V3 ESBL culture.pdf DRUM_SOP4_V2 K. pneumoniae identification.pdf DRUM_SOP5_V2 Storage.pdf Data are available under the terms of the Creative Commons Attribution 4.0 International license (CC-BY 4.0).
