# Supplementary material for: Drivers of Resistance in Uganda and Malawi (DRUM): a protocol for the evaluation of One-Health drivers of Extended Spectrum Beta Lactamase (ESBL) resistance in Low-Middle Income Countries (LMICs)
Source: Wellcome Open Res. 2023 May 2;7:55. Originally published 2022 Feb 15. [Version 2] doi: 10.12688/wellcomeopenres.17581.2 (PMC11137479; doi:10.12688/wellcomeopenres.17581.2)
Supplement: Peer review history

# Reviewer report: Reviewer response for version 2

**DOI:** 10.21956/wellcomeopenres.21495.r89559

**Published:** 2024-09-03

**Authors:** Mushtak T.S. Al-Ouqaili

**Affiliations:** 1 College of Medicine, University of Anbar, Al-Anbar, Iraq

Dear authors,

 Hope you are doing well and that’s look great work. A few issues, however, need to be addressed;

 Your introduction is comprehensive and provides a clear overview of the significance of antimicrobial resistance (AMR), particularly in sub-Saharan Africa (sSA), and the objectives of the DRUM Consortium's study in Uganda and Malawi.

**Here are some suggestions to enhance the introduction:**

- Incorporate recent statistics or findings on the prevalence of ESBL-producing bacteria in sSA, Uganda, and Malawi. This will underscore the urgency and relevance of the study. Add the following reference: Al-Ouqaili *et al.*, 2023 ^1^.
- Emphasize the specific knowledge gaps that the study aims to fill, particularly regarding the transmission routes of ESBL-producing bacteria. Add the following reference: Owaid and Al-Ouqaili, 2024 ^2^.
- Explicitly state the primary goals of the study and how the findings will contribute to addressing AMR. For example, you can mention specific interventions that might be developed based on the study's outcomes.

**Site 1: Ndirande, Blantyre, Malawi (Urban)**

 Your description of the Malawian site in Ndirande, Blantyre is detailed and informative. However, there are a few areas where additional information could be beneficial:

- Include specific data on population density or housing density to give a clearer picture of the urban environment.
- Provide more details about the Ndirande Health Centre and Queen Elizabeth Central Hospital (QECH), such as the services they offer, their capacity, and their roles in the healthcare system.
- Highlight any environmental factors that may impact the spread of infectious diseases, such as seasonal variations, climate conditions, or pollution.

**Site 2: Chileka, Blantyre, Malawi (Peri-Urban):**

- Provide specific data on the population size, density, and demographic composition (e.g., age distribution, household size).
- Briefly mention the main economic activities or sources of livelihood for residents in Chileka, as these can influence health and antibiotic use.

**Site 3: Chikwawa, Malawi (Rural)**

 The description of Chikwawa as a rural site in Malawi is detailed and informative. To enhance it further, consider adding the following information:

- Provide specific data on the demographic composition of Chikwawa, including age distribution and household size.
- Include more details about Chikwawa District Hospital, such as the types of services offered, capacity, and its role within the district’s healthcare system.

**Ugandan site descriptions**

**Site 4: Kampala, Uganda (Urban)**

 The description of the Kampala site in Uganda provides a good starting point. To make it more comprehensive, consider adding the following information:

- Mention any ongoing public health initiatives or programs in Kampala related to AMR, malaria, or infectious diseases.
- Describe the transportation infrastructure and its impact on access to healthcare facilities, especially during emergencies or peak times.

**Methods**

 The methods section on the household selection process is detailed and informative. Here are a few suggestions for additional information that could enhance the description:

- Explain why the inhibitory-with-close-pairs approach is suitable for studying AMR transmission specifically, and how it compares to other potential sampling methods.
- More details about the study areas' geospatial characteristics (e.g., urban layout, rural dispersion) can help clarify the rationale behind the chosen distances (MID and CPR).
- Mention any ethical considerations, including informed consent procedures and how participants' privacy and data confidentiality are maintained.

**Participant eligibility**

 The participant eligibility criteria are clear and well-defined. To make the description more comprehensive, consider adding the following information:

- Specify any additional household characteristics that are considered for eligibility, such as household size, age distribution, or specific living conditions.

**Data collection**

**Case Report Forms (CRFs)**

 The data collection section outlines the design and implementation of Case Report Forms (CRFs) effectively. To make this description more comprehensive, consider adding the following information:

- Describe the process of designing the CRFs, including any pilot testing or validation steps to ensure their effectiveness and reliability.
- Provide details on the tools and techniques used for data collection, such as paper-based forms, electronic data capture systems, or mobile applications.

**Microbiological Methodology**

 The microbiological methodology section is detailed and thorough. To ensure completeness and clarity, consider adding the following information:

- Describe any quality control procedures to ensure the accuracy and reliability of the microbiological methods, such as the use of controls or validation steps.
- Provide details on the handling and transport of samples from the field to the laboratory to ensure sample integrity and prevent contamination.
- Explain how data from microbiological analysis will be recorded, managed, and analyzed.

**DNA extraction, Library preparation and Sequencing**

- Describe any quality control steps during DNA extraction to ensure the purity and integrity of extracted DNA.
- Provide more information on the specific steps involved in library construction using the NEB Ultra II custom kit and sequencing platforms such as Illumina X10, MinION, and HiSeq 4000.

**Community engagement and involvement**

 To enhance the section on community engagement and involvement, consider including the following details:

- Clearly state the objectives of community engagement, such as fostering trust, obtaining community input, and ensuring transparency throughout the study.
- Describe specific methods used to engage with the community, such as community advisory groups, meetings with local leadership, and involvement of district health offices and councils.

Conclusion should be objective with further perspective or should add at least a few sentences about future study/future perspective of it.

Is the study design appropriate for the research question?

Yes

Is the rationale for, and objectives of, the study clearly described?

Yes

Are sufficient details of the methods provided to allow replication by others?

Yes

Are the datasets clearly presented in a useable and accessible format?

Yes

Reviewer Expertise:

Medical Microbiology including immunology, bacteriology in addition to molecular biology

I confirm that I have read this submission and believe that I have an appropriate level of expertise to confirm that it is of an acceptable scientific standard.

## References

[1] : Study of vacuolating cytotoxin A (vacA) genotypes of ulcerogenic and non-ulcerogenic strains of Helicobacter pylori and its association with gastric disease. *Saudi J Biol Sci* .2023;30(12) : 10.1016/j.sjbs.2023.103867 103867 10.1016/j.sjbs.2023.103867 38020230 PMC10663908

[2] : Molecular and bacteriological investigations for the co-existence CRISPR/Cas system and β-lactamases of types extended-spectrum and carbapenemases in Multidrug, extensive drug and Pandrug-Resistant Klebsiella pneumoniae. *Saudi J Biol Sci* .2024;31(7) : 10.1016/j.sjbs.2024.104022 104022 10.1016/j.sjbs.2024.104022 38817398 PMC11137337

# Reviewer report: Reviewer response for version 2

**DOI:** 10.21956/wellcomeopenres.21495.r82567

**Published:** 2024-05-30

**Authors:** Emily K. Rousham

**Affiliations:** 1 Centre for Global Health and Human Development, School of Sport, Exercise and Health Sciences, Loughborough University, Loughborough, UK

This protocol describes a longitudinal study of the presence of antibiotic resistant organisms in humans, animals and the environment in Uganda and Malawi using a One Health approach. Data will be collected on human behaviours, household water, sanitation and hygiene infrastructure, socio-demography, microbiology and genomics to gain interdisciplinary insights on drivers of AMR. The aim is to create agent-based models to describe the transmission of extended spectrum beta-lactamase (ESBL) resistance in *E. coli* and *K. pneumoniae*.

 It is a large scale study of its type and will provide valuable datasets. The multidisciplinary approach adds further value to the research. The extended data provide a comprehensive resource.

 Some suggestions to facilitate replication are indicated below.

 Methods, Recruitment of Households

 A rationale for the sample size of 100 households per site (500 in total) would be a valuable addition. How was this determined?

 Participant eligibility: The eligibility criteria are residing within the study site (polygon) and being willing to provide a minimum 12 samples at baseline. It is implicit that animal ownership was not a criterion for inclusion, but it may be helpful for readers to state this explicitly (i.e. households with or without animals were eligible), should the authors wish to.

 It would be useful to state the minimum and maximum number of samples to be collected per household per visit in the same sentence. At present ‘Participant eligibility’ states ‘a minimum of 12 samples at baseline’ and ‘Longitudinal microbiological sampling’ states ‘a maximum of 20 samples per visit, inclusive of human and animal stool samples and environmental samples’.

 Across the 12 to 20 samples collected per household, other than the minimum of 2 samples from humans, were there targets or a minimum set for the number of animal (where applicable) and environmental samples?  

 The list of companion and livestock animals in the standard operating procedure for animal stool sampling could be provided in the text for easy reference.

 Field sampling methods: after ‘Human stool will be self-collected in a 30 mL stool pot by participants’… it may be helpful to add  ‘or via rectal swab for those unable to provide a stool sample’ if this was the case as indicated from the standard operating procedure.

 Household individuals were eligible for inclusion if they did not have confirmed or suspected acute bacterial infection at the time of recruitment. If a recruited individual had no bacterial infection at illness at baseline, but had a bacterial infection at a follow-up visit, were they excluded just for that sample collection, or for all subsequent (or previous) sample collections?

 The procedures for consent could be expanded to cover participants aged under 18 years (if applicable). For the individual level surveys, the age at which participants responded to the survey themselves (if under 18 year olds were recruited) could be stated along with the ages where another person responded on behalf of the participant.

 Understanding WASH behavioural drivers: some aspects of the RANAS component were not entirely clear without knowing the household contexts. What proportion of households in the study sites have household staff members, and could the terms ‘doers’ and ‘non-doers’ be explained?

 Minor typographical suggestions:

 Abstract, line 16: in the context of this sentence, ‘complemented’ may be more appropriate.

 Malawian site descriptions: the sentence ‘Healthcare is free at the point of delivery in Uganda and Malawi, and this should be assumed unless otherwise stated’ would be better placed in the paragraph above, since it applies to both countries not just Malawi.

 Study status section, final sentence: should this be ‘undergoing’?

Is the study design appropriate for the research question?

Yes

Is the rationale for, and objectives of, the study clearly described?

Yes

Are sufficient details of the methods provided to allow replication by others?

Partly

Are the datasets clearly presented in a useable and accessible format?

Not applicable

Reviewer Expertise:

Population health, community-based interventions, interdisciplinary approaches, global health, evidence synthesis.

I confirm that I have read this submission and believe that I have an appropriate level of expertise to confirm that it is of an acceptable scientific standard.

# Reviewer report: Reviewer response for version 1

**DOI:** 10.21956/wellcomeopenres.19440.r52673

**Published:** 2022-10-26

**Authors:** Katarina Oravcova

**Affiliations:** 1 School of Biodiversity, One Health and Veterinary Medicine, University of Glasgow, Glasgow, UK

**Article summary:**

 This manuscript by Cocker *et al.* describes a study protocol to investigate the drivers of antimicrobial resistance in two sub-Saharan countries. The study protocol focuses on the AMR representative extended spectrum beta-lactamase (ESBL) producing organisms. The protocol is a comprehensive One Health study employing a variety of data and samples from 400 households in Uganda and Malawi, and interdisciplinary research approaches to pinpoint the drivers of resistance, from human behaviour, through antibiotic use, WASH practices, and genomic epidemiology investigation of human, animal and environmental carriage of ESBL producers.

 The background and motivation for this study are clearly stated and well justified. The protocol is clearly presented and logically structured. The range of methods is described with very good level of detail. Overall, this study protocol is bound to generate multidisciplinary data of significant quality and quantity, which in turn will contribute to improved understanding of the drivers of AMR and unravel potential ways of tackling these.

 I have the following comments addressing a few specific points relevant to article format “study protocol” requirement of “sufficient details of the methods provided to allow replication by others”:

 One of the motivations for this study was the burden due to “severe bacterial infection” “compounded by antimicrobial resistance”. Please describe how your study of carriage (from healthy individuals) and environmental isolates will link to clinical isolates and understanding of resistance transmission to/from/within clinical settings.

 Genomic analyses will be done predominantly on culture-selected ESBL producing organisms. Is AMR and multidrug resistance going to be established by phenotypic antimicrobial susceptibility testing of the isolates? If so, is AST going to be evaluated with the identified genomic determinants of resistance? If not, why not? Please specify and/or add this in the protocol.

**Further specific comments:**

Introduction:

- Statement “The 3rd-generation cephalosporin (3GC) ceftriaxone is frequently the antimicrobial agent of first and last resort across much of sSA.” Modify this statement so that you refer to 3 ^rd^ generation cephalosporins as a class, rather than a single drug, or expand and explain why ceftriaxone is “of first and last resort across much of sSA” and support it with a reference.
- Add “genes encoding” in the statement “…due to acquisition of genes encoding extended-spectrum beta lactamase (ESBL)-producing enzymes…”.
- What is the incidence of neonatal sepsis and malaria? What is the prevalence of HIV in these countries?
- Consider modifying this sentence to “…focus on isolation and genomic characterisation of ESBL producing *E. coli *(ESBL-E) and *K. pneumoniae *(ESBL-K).”
- Specify if in the statement “We will take an interdisciplinary, One-Health approach to assess how human behaviour, WASH practices, environmental contamination,…” you are referring to “environmental contamination by enteric bacteria” or other.
- In the final sentence “…on interrupting transmission.” specify if you are referring to bacterial transmission, resistance transmission, or other.

Site selection:

- Why were there three sites defined in Malawi (sites 1, 2 and 3) but only two sites (sites 4 and 5) in Uganda? Was it due to the contiguous location of site 5 in Uganda? How is this going to be considered for future comparative data analysis (i.e. 100 households per site)?

- Consider using “animal husbandry practices” instead of “animal practices”, or just “husbandry practices” as farming areas are mentioned in Fig. 2.
- Do you refer to “environmental contamination” (of household or premises?) in “contamination with ESBL-producing bacteria”? Please specify this.
- Was the socioeconomic status of the sites in Kampala and Blantyre comparable? Is there information on housing density comparison between the equivalent sites in Uganda and Malawi? Can you expand on this a bit more?
- Did you mean “Blantyre, the second largest city of Malawi”?
- What was the estimated population of Chileka?
- What was the estimated population of the urban study site in Kampala?
- Site 5: Hoima – check the subheading and the text in this paragraph. Was Hoima town area defined as urban (subheading) or peri-urban (in the text)?

Methods:

- How was the “apparent uniformity of the population density across the study area” estimated in Kampala and Chileka?
- How was the data on socioeconomic gradient acquired for "In Kampala, the availability of a marked socioeconomic gradient within the study region”? Please use a reference if possible.
- In the statement “… and not have confirmed or suspected acute infection”, does this take HIV status into account?
- Figure 3 – please clarify if sampling procedure in Fig. 3 or in the main text is preferred, i.e. will 3-4 visits be done within 6 months (Fig) or 12 months (text); which is more representative of seasonality? Malawi has one rainy season, Uganda has two rainy seasons.
- In the statement “…sites that serve as important reservoirs of ESBL AMR.” – did you mean “ESBL-producing organisms”?
- Household environmental sampling – what size of surfaces were sampled and/or what exposure time was used for sampling (e.g. for boot socks)? Can you specify this please?
- Microbiological methodology order – to improve the flow of this section, specify pre-processing steps at first (e.g. filtration, centrifugation, homogenization etc.), then describe pre-enrichment (in BPW if needed), and then culture and isolation.
- Write out abbreviations in full at first use, e.g. buffered peptone water (BPW).
- Drain samples – specify if these are inner drains (e.g. in the kitchen or hand washing sinks) or outer drains (sewage, other).
- Manufacturers – manufacturers are written out for some consumables (e.g. VWR for water filtering, sampling bags) but not for other consumables. For consistency, decide on including all products and manufacturers or make these generic.
- Consistency in fruit and vegetable preparation for culture – what ratios and volumes of fruit and BPW will be used? Will peel of fruits be included for culture? What mass/weight of green leafy vegetables will be used?
- Terminology – choose either “massaged” or “manually stomached” if these refer to the same processes.
- Please check clarity of this sentence “Green leafy vegetables will be weighed and have nine times the weight of the food added in BPW to obtain a sample-to-diluent volume of 1:9”. Do you refer to weights or volumes? Did you mean the diluent (BPW) will have 9 times the weight/volume of the leafy vegetables?
- Storage of BPW cultures at -80°C – is any glycerol going to be added?
- Inoculation on CHROMagar – what volume of samples cultured in BPW will be plated on the selective agar? It was not specified what volume of BPW would be used for pre-enrichment.
- Plate sweeps – these are mentioned for the first time after ESBL isolates. What cultures and plates will be used for plate sweeps, the same as those for isolated ESBL producers?
- ESBL isolation – how many suspected ESBL colonies will be selected from each sample? A single colony of each ESBL-E and ESBL-K if present or multiple?
- Original sample storage for metagenomics – how will these be stored? E.g. will you store filtered and concentrated water, will you store swabs or swabs homogenized in PBS?
- It is not necessary to mention metagenomic tools (mSWEEP/mGEMS) in sample storage section, and it would be more suitable for sequencing data analysis section.
- DNA extraction and sequencing – how will ESBL-negative isolates be acquired and selected? This should be specified in the microbiology section.
- What library preparation kit will be used for Illumina sequencing?
- Numbers of samples for metagenomic sequencing are specified. How many isolates and sweeps will be whole genome sequenced on an Illumina platform? How many or up to how many isolates will be Nanopore sequenced?
- Metagenomic resistome of PBP pre-enriched samples will be investigated. Will you investigate the resistome of CHROMagar plate sweeps? If so, please specify this.
- Spatial analysis and integration of datasets into an agent-based model. ***“*** The initial approach will be to determine variables (as described above)” please specify in which paragraph are the variables mentioned. While biological sampling and microbiological analysis are described in a great level of detail, this section is less evolved. What software, computational tools will be used? What geostatistical models will be used? How will qualitative components be evaluated and chosen for ABM?
- As this manuscript is a study protocol and investigating “the transmission of ESBL-E and ESBL-K between humans, animals, and the environment” was one of the study objectives, it would be valuable to add a section on genomic epidemiological methods to investigate the transmission (although, this may be further detailed in publication of the study results).

Is the study design appropriate for the research question?

Yes

Is the rationale for, and objectives of, the study clearly described?

Yes

Are sufficient details of the methods provided to allow replication by others?

Partly

Are the datasets clearly presented in a useable and accessible format?

Not applicable

Reviewer Expertise:

microbiology, bacterial pathogens, antimicrobial resistance, ESBL, TB, Streptococcus, WGS, genomics, East Africa

I confirm that I have read this submission and believe that I have an appropriate level of expertise to confirm that it is of an acceptable scientific standard, however I have significant reservations, as outlined above.

# Response

**Published:** 2023-04-17

**Authors:** Derek Cocker

**Affiliations:** Liverpool School of Tropical Medicine, UK

**Reviewer 2:** **Article summary:** **I have the following comments addressing a few specific points relevant to article format “study protocol” requirement of “sufficient details of the methods provided to allow replication by others”:**

**One of the motivations for this study was the burden due to “severe bacterial infection” “compounded by antimicrobial resistance”. Please describe how your study of carriage (from healthy individuals) and environmental isolates will link to clinical isolates and understanding of resistance transmission to/from/within clinical settings.** The study aims to understand the transmission of bacteria with known human pathogenic potential in the community. It will be conducted in two settings with robust capacity to undertake surveillance of severe bacterial infection in individuals from the study sites. MLW, Malawi has offered quality assured diagnostic microbiology services to patients presenting to Queen Elizabeth Central Hospital, Blantyre with suspected sepsis, an area including Ndirande and Chileka since 1998 and more recently to febrile patients presenting to Chikwawa district hospital through the FIEBRE study (Hopkins H et al, BMJ Open. 2020 Jul 21;10(7):e035632), which was contemporaneous to DRUM. IDI has secured  grant funding to undertake bacteraemia surveillance in Uganda as it was a Fleming Fund inception site (https://www.flemingfund.org/countries/uganda/). The link to clinical samples will therefore be made through whole genome sequencing of isolates both from DRUM and from the archives of these clinical diagnostic services. We will not undertake resistance transmission within clinical settings as this is not a study of healthcare associated infection. **Genomic analyses will be done predominantly on culture-selected ESBL producing organisms. Is AMR and multidrug resistance going to be established by phenotypic antimicrobial susceptibility testing of the isolates? If so, is AST going to be evaluated with the identified genomic determinants of resistance? If not, why not? Please specify and/or add this in the protocol.** The genomic analysis will be undertaken on a combination of culture-selected ESBL producing organisms and enrichment broth. For isolates growing on ESBL-selective media, we will not formally confirm ESBL status, rather this will be inferred from growth on ESBL CHROMagar™ media. This decision was taken because we have previously validated ESBL CHROMagar™ media in our laboratory in Malawi for this purpose (manuscript in preparation). During this validation exercise, ( *J. Lewis, personal communication*) 663 ESBL Enterobacteriaceae isolates that had screened positive by ESBL CHROMagar methods had ESBL phenotype formally confirmed by combination disc testing, as per EUCAST guidance. Of the 663 bacterial isolates included in this validation exercise, ESBL production was formally confirmed phenotypically in 659/663 (99.4%). Specifically, 447/449 (99.55%) for ESBL- *E. coli* and 156/156 (100%) for ESBL- *K. pneumoniae*. Having established there was minimal to be gained from formal phenotypic confirmation and given our we plan to undertake molecular confirmation of ESBL status of isolates growing on 3 ^rd^-generation cephalosporin containing media (and therefore phenotypically resistant to 3 ^rd^-generation cephalosporins) by whole genome sequencing we decided against formal confirmation of ESBL status due to time and cost.   Aside from ESBL screening, phenotypic antimicrobial susceptibility testing will not be undertaken, and will instead be inferred from the genotype.   We have noted the limitation that phenotypic confirmation of ESBL status will not be made, by  amending the manuscript to clearly state that “ *ESBL status will be inferred by chromogenic methods alone and not formally confirmed by phenotype, however isolates growing on ESBL media will be whole genome sequenced.* *”* **Further specific comments:** **Introduction:**

- **Statement “The 3rd-generation cephalosporin (3GC) ceftriaxone is frequently the antimicrobial agent of first and last resort across much of sSA.” Modify this statement so that you refer to 3 ^rd^ generation cephalosporins as a class, rather than a single drug, or expand and explain why ceftriaxone is “of first and last resort across much of sSA” and support it with a reference.**

This comment was noted by Reviewer 1. We have now reworded the statement to read that *“* *The 3 ^rd^-generation cephalosporin (3GC) ceftriaxone is frequently the antimicrobial agent of choice* *for the empirical management of sepsis across much of sSA”* and referenced papers on its use in one of the study states (Malawi) and globally. References addition (Lester et al. Clin Infect Dis. 2020 Dec 3;71(9):e478–86) & Browne AJ et al. Lancet Planet Health. 2021 Dec;5(12):e893-e904)

- **Add “genes encoding” in the statement “…due to acquisition of genes encoding extended-spectrum beta lactamase (ESBL)-producing enzymes…”.**

This wording has now been added into the manuscript, as follows: *“The 3 ^rd^-generation cephalosporin (3GC) ceftriaxone is frequently the antimicrobial agent of first choice for the empirical management of sepsis across much of sSA* *(6-7)* *. 3GC resistant (3GC-R) enteric bacteria have rapidly emerged, largely due to acquisition of genes encoding extended-spectrum beta lactamase (ESBL)-producing enzymes, resulting in infections that are frequently locally untreatable, due to unavailability of carbapenems or other reserve antibiotics”*

- **What is the incidence of neonatal sepsis and malaria? What is the prevalence of HIV in these countries?**

The wording of this sentence has now been changed to reflect the burden of disease rather than incidence of sepsis and Malaria, as per comments by reviewer 1. We have amended the manuscript to state that there is a “high burden of disease from neonatal sepsis and malaria”. The details of this are included in the references attached for maternal sepsis ( *Seale AC et al. Maternal and early onset neonatal bacterial sepsis: burden and strategies for prevention in sub-Saharan Africa. Lancet Infect Dis. 2009 Jul;9(7):428–38*) and malaria (WHO. World Malaria report. Geneva, World Health Organization, Global Malaria Programme; 2021.). We have now included data on the burden of malaria in the site sections for Malawi *(“* *According to the Malawian Health Management Information System report, malaria accounts for about 34% of all outpatient visits and 18% of all hospital deaths in Malawi* *(22)* *”*) and Uganda *(“ In 2020, Uganda had the 3rd highest global burden of malaria cases and deaths (5.4%) and the 5th highest level of deaths (3.5%)* *(13)*). In relation to the HIV prevelance in Malawi and Uganda, this has already been stated in the text, with associated references in the study site sections, as follows: (Malawi: “HIV prevalence in adults aged 15–65 is 18% and there is a high burden of typhoid and tuberculosis (17,18).”. Kampala: “Adult HIV prevalence is 6.9% (21).”, Hoima: “HIV prevalence among adults aged 15–64yrs in the Mid-West Region of Uganda where Hoima is located is 5.7% (21).”) 

- **Consider modifying this sentence to “…focus on isolation and genomic characterisation of ESBL producing *E. coli *(ESBL-E) and *K. pneumoniae *(ESBL-K).”**

Thank you for the comment. We have amended the wording of this statement thus; *“DRUM will work in urban, peri-urban, and rural settings in Uganda and Malawi and focus on the isolation and genomic characterisation of ESBL producing E. coli (ESBL-E) and K. pneumoniae (ESBL-K).”*

- **Specify if in the statement “We will take an interdisciplinary, One-Health approach to assess how human behaviour, WASH practices, environmental contamination,…” you are referring to “environmental contamination by enteric bacteria” or other.**

We have now amended to specify contamination with ESBL bacteria, as follows: *“We will take an interdisciplinary, One-Health approach to assess how human behaviour, WASH practices, environmental contamination with ESBL bacteria, and ABU in urban and rural locations within Uganda and Malawi contribute to the transmission of ESBL-E and ESBL-K between humans, animals, and the environment and how this transmission relates to strains isolated from the blood of humans with drug-resistant infection (DRI).”*

- **In the final sentence “…on interrupting transmission.” specify if you are referring to bacterial transmission, resistance transmission, or other.**

We have amended to specify bacterial transmission, as follows: *“We will collect demographic, geospatial, WASH, longitudinal clinical and molecular microbiological data, and integrate these data into agent-based models designed to estimate the impact of putative interventions on interrupting transmission of ESBL bacteria.”* **Site selection:**

- **Why were there three sites defined in Malawi (sites 1, 2 and 3) but only two sites (sites 4 and 5) in Uganda? Was it due to the contiguous location of site 5 in Uganda? How is this going to be considered for future comparative data analysis (i.e. 100 households per site)?**

Yes, it was indeed due to the characteristics of site 5 in Uganda, which had both peri-urban and rural characteristics, and peri-urban and rural polygons within Hoima were classified a priori. **Consider using “animal husbandry practices” instead of “animal practices”, or just “husbandry practices” as farming areas are mentioned in Fig. 2.** This has been amended within the manuscript to animal husbandry practices, as follows: *“DRUM consortium members identified sites representing urban, peri-urban, and rural settings to enable variations in WASH behaviours, animal husbandry practices, ABU, and household and broader environmental contamination with ESBL-producing bacteria to be contrasted.”*

- **Do you refer to “environmental contamination” (of household or premises?) in “contamination with ESBL-producing bacteria”? Please specify this.**

We have amended the wording to be more specific, including household and broader environmental contamination, as follows: *“DRUM consortium members identified sites representing urban, peri-urban, and rural settings to enable variations in WASH behaviours, animal husbandry practices, ABU, and household and broader environmental contamination with ESBL-producing bacteria to be contrasted.”*

- **Was the socioeconomic status of the sites in Kampala and Blantyre comparable? Is there information on housing density comparison between the equivalent sites in Uganda and Malawi? Can you expand on this a bit more?**

The socioeconomic status of Kampala and Blantyre is broadly comparable, but there will be variation between the  households, which will be quantified in numerous ways in this study using the household questionnaires; details of which are included in the household CRFs (questions 15-22). We have highlighted in the text that socioeconomic status will be evaluated in the questionnaires. *“At the baseline visit, these CRFs will be completed to provide information at the individual and household level on human health, ABU, socioeconomic status, health seeking behaviour, structural and behavioural WASH practices and animal husbandry (Extended data).” & Table 1.  * We do not have specific data on housing density at either site and this has not been included as a variable in our analyses.

- **Did you mean “Blantyre, the second largest city of Malawi”?**

Thank you for this comment. This has now been amended to make it clearer, as recommended. Now stating in the manuscript that Blantyre, the second largest city of Malawi. *“Ndirande is a large urban settlement with high-density housing 4 km from the geographical centre of Blantyre, the second largest city of Malawi* *(13,14)* *”*

- **What was the estimated population of Chileka?**

The estimated population of Chileka is 34,720. This figure has been obtained via verbal correspondence with the Blantyre District Health Office, as it is not available through published sources.

- **What was the estimated population of the urban study site in Kampala?**

The areas sampled comprise of a number of neighbourhoods within Rubaga Division of Kampala District. Rubaga has an estimated population of 427,300 (1). (1) https://www.citypopulation.de/en/uganda/central/admin/kampala/SC0479__rubaga/

- **Site 5: Hoima – check the subheading and the text in this paragraph. Was Hoima town area defined as urban (subheading) or peri-urban (in the text)?**

Hoima has two polygons, comprising of a peri-urban area and rural area. This has now been amended in the text and subheading for consistency. **Methods:**

- **How was the “apparent uniformity of the population density across the study area” estimated in Kampala and Chileka?**

The uniformity of population density was estimated through visual inspection of OpenStreetMap building location data plotted over satellite imagery of each area.

- **How was the data on socioeconomic gradient acquired for "In Kampala, the availability of a marked socioeconomic gradient within the study region”? Please use a reference if possible.**

No formal data describing socioeconomic metrics of these specific regions of Kampala exist. Socioeconomic gradients for areas included within the polygons were determined based on local expert opinion, via information obtained through verbal communication with local health teams and WASH organisations, and considered alongside broad geographic details included in the 2013 Uganda “slum settlement profile” (https://askyourgov.ug/).

- **In the statement “… and not have confirmed or suspected acute infection”, does this take HIV status into account?**

To clarify, this is to reduce the chance that our participants have an active bacterial infection at recruitment and is not specifically related to HIV status. We have, therefore, amended this in the text, as follows: *“and not have confirmed or suspected acute bacterial infection at the time of recruitment.”*

- **Figure 3 – please clarify if sampling procedure in Fig. 3 or in the main text is preferred, i.e. will 3-4 visits be done within 6 months (Fig) or 12 months (text); which is more representative of seasonality? Malawi has one rainy season, Uganda has two rainy seasons.**

The follow-up for each household (and associated sampling) will occur at 3-4 visits over a 6-month period. This is reflected in Figure 2 and in the text. We will, however, stagger recruitment of the 100 households per region over a 12-month period (i.e. ~8-10 households per region, per month), and this will enable us to have baseline (and follow-up) data from houses that span the period of a year, enabling us to capture data in wet and dry seasons for both Malawi and Uganda.  

- **In the statement “…sites that serve as important reservoirs of ESBL AMR.” – did you mean “ESBL-producing organisms”?**

Thank you for this comment. We have reworded the manuscript, as follows: *“We decided to focus on areas identified as hand-contact zones or where food handling occurred and also to include broader environmental sites that we hypothesise serve as important reservoirs of ESBL-producing organisms.”*

- **Household environmental sampling – what size of surfaces were sampled and/or what exposure time was used for sampling (e.g. for boot socks)? Can you specify this please?**

Thank you for this comment. We have now included the collection SOPs, which provide details of all environmental sampling methods inclusive of boot socks and household environmental swabbing in the extended material (https://doi.org/10.5281/zenodo.7561679). To answer the reviewer’s specific questions, the size of the surface for household environmental swabs was 30cm ^2^ and boot socks were worn for 1 minute. These are described in detail in newly added SOPs.

- **Microbiological methodology order – to improve the flow of this section, specify pre-processing steps at first (e.g. filtration, centrifugation, homogenization etc.), then describe pre-enrichment (in BPW if needed), and then culture and isolation.**

The order of this section has now been changed to reflect this flow.

- **Write out abbreviations in full at first use, e.g. buffered peptone water (BPW).**

For reference, BPW was first mentioned in the field sampling section, so no amendments were made in relation to abbreviations for BPW. The rest of the document has been checked for abbreviation use, and we have added in the full title for SOP prior to its use. ANOVA and HIV remain in the manuscript text having not been expanded upon, which is consistent with other journal manuscripts.

- **Drain samples – specify if these are inner drains (e.g. in the kitchen or hand washing sinks) or outer drains (sewage, other).**

Thank you for this comment. We have now stated as “Outer drains” in the text to clarify their location. Also we have added collection SOPS to the protocol (https://doi.org/10.5281/zenodo.7561679), so these methods are further clarified.

- **Manufacturers – manufacturers are written out for some consumables (e.g. VWR for water filtering, sampling bags) but not for other consumables. For consistency, decide on including all products and manufacturers or make these generic.**

We have now added the manufactures onto all products stated in the body of the text for consistency. Updates include BPW (Oxoid) and HRM-PCR (Qiagen).

- **Consistency in fruit and vegetable preparation for culture – what ratios and volumes of fruit and BPW will be used? Will peel of fruits be included for culture? What mass/weight of green leafy vegetables will be used?**

We feel this is adequately stated in the text and expanded on within the extended material and SOPs. The ratio of 1:9 sample-diluent only pertains to green leafy vegetables, whereas fruit will have enough BPW added to cover. Our focus for fruit pertains to the outside, where hand-contact or environmental contact with AMR bacteria can take place. In this regard a whole fruit is used. We have stated “whole” in the methods section for clarification, and also added the collection SOPs where it states that fruit are to be sampled whole (without peel removed for citrus samples). The mass of vegetables used can vary and is not a set amount. By using a consistent 1:9 sample-diluent approach this will standardise the processing between various vegetables recovered. These approached were designed in conjunction with specialist advice from the UK Health Security Agency Food Water and Environment (FWE) Microbiology Services.

- **Terminology – choose either “massaged” or “manually stomached” if these refer to the same processes.**

These refer to a similar process, so we have made this more consistent in the manuscript as follows: *“Fruit (whole) will have enough BPW added to the Whirl-Pak® bag to cover before being manually stomached for a period of 30 sec to 3 min.”*

- **Please check clarity of this sentence “Green leafy vegetables will be weighed and have nine times the weight of the food added in BPW to obtain a sample-to-diluent volume of 1:9”. Do you refer to weights or volumes? Did you mean the diluent (BPW) will have 9 times the weight/volume of the leafy vegetables?**

We have amended the wording to clarify this, as follows: *“Green leafy vegetables will be weighed and have nine times the weight of the food added in BPW (Oxiod) to obtain a sample-to-diluent weight ratio of 1:9”*

- **Storage of BPW cultures at -80°C – is any glycerol going to be added?**

For BPW (enrichment broth), these are stored at -80°C without the addition of glycerol. Glycerol is added to BPW stock, used for the storage of ESBL isolates. The details of this process are included within laboratory SOPs 1,2 and 3. We store the BPW directly (without glycerol), as DNA extraction will occur only once, whereas the isolates may need to be re-plated or used for future analysis.

- **Inoculation on CHROMagar – what volume of samples cultured in BPW will be plated on the selective agar? It was not specified what volume of BPW would be used for pre-enrichment.**

We have amended to state a “1µl loop” will be plated onto ESBL CHROMagar™. The details for this are also in the extended material under ESBL culture SOP.

- **Plate sweeps – these are mentioned for the first time after ESBL isolates. What cultures and plates will be used for plate sweeps, the same as those for isolated ESBL producers?**

To clarify, the plate sweeps will occur directly from the CHROMagar plates where there is growth of ESBL colonies (as per our SOPs in the extended material). We have now added an additional line to clarify this prior to the storage paragraph, as follows: *A plate sweep will be taken from each ESBL CHROMagar™ plate that has positive growth of ESBL colonies.*

- **ESBL isolation – how many suspected ESBL colonies will be selected from each sample? A single colony of each ESBL-E and ESBL-K if present or multiple?**

Details of this are included in Section 4.4 of laboratory SOP3. A single pick will be taken of pink, blue and white colonies and white colonies/ blue colonies will undergo further additional testing. Most white colonies are not ESBL *E. coli*, but we know from local experience that this is a possibility, and so we test them to ensure that they do not represent false negatives in our phenotypic results. If however they were to be indole positive, we would store them as well. In this regard, we will take between 1-2x ESBL-E and 1x ESBL-K per plate.

- **Original sample storage for metagenomics – how will these be stored? E.g. will you store filtered and concentrated water, will you store swabs or swabs homogenized in PBS?**

The only direct sample that is stored unprocessed is human and animal stool (laboratory SOP 1, section 4.3). Duplicate river water (filtrate membranes) are also stored (laboratory SOP2, section 4.4.3) representing concentrates of the bacteria present in river water. No PBS is added to these membranes, as recommended by specialist advice from environmental scientists at CEH. For other sample types (i.e. environmental swabs, boot socks, food etc) only the BPW diluent will be stored. Details of these processes are included in the lab SOPs 1/2.

- **It is not necessary to mention metagenomic tools (mSWEEP/mGEMS) in sample storage section, and it would be more suitable for sequencing data analysis section.**

As there is a specific purpose of storage, and storage can impact on findings at subsequent metagenomic genomic analysis (i.e. diversity and relative abundance of species) we have retained this information here.

- **DNA extraction and sequencing – how will ESBL-negative isolates be acquired and selected? This should be specified in the microbiology section.**

As we are no longer planning on sequencing ESBL-negative isolates in the primary phase of our genomic analysis we have removed the line where this is stated in this protocol. As stated in our SOPS, aliquots of buffered peptone water, which are not under antimicrobial selection, will be stored at -80˚C, and if necessary, these will be regrown on MacConkey Agar to obtain ESBL-negative isolates at a later date. Any details of the methods employed in selection and regrowth of ESBL-negative isolates will be included in subsequent papers, should this be work be undertaken.

- **What library preparation kit will be used for Illumina sequencing?**

We have added details on library preparation, including kits used to the DNA extraction in the library preparation and sequencing section.

- **Numbers of samples for metagenomic sequencing are specified. How many isolates and sweeps will be whole genome sequenced on an Illumina platform? How many or up to how many isolates will be Nanopore sequenced?**

We have added the number of plate sweeps (1500) but are unable to specify the number of single colony pick isolates we will sequence on Illumina platform as we plan to sequence all ESBL positive isolates, and thus the final number is dependent on the ESBL positivity rate.  Similarly, we are unable to specify the number of isolates to be sequenced on nanopore, as these will be representative of the major lineages we will identify and therefore dependent on the diversity of the of isolate whole genomes which we do not know beforehand.

- **Metagenomic resistome of PBP pre-enriched samples will be investigated. Will you investigate the resistome of CHROMagar plate sweeps? If so, please specify this.**

Yes, and a line has been added to reflect this.

- **Spatial analysis and integration of datasets into an agent-based model. *“*The initial approach will be to determine variables (as described above)” please specify in which paragraph are the variables mentioned. While biological sampling and microbiological analysis are described in a great level of detail, this section is less evolved. What software, computational tools will be used? What geostatistical models will be used? How will qualitative components be evaluated and chosen for ABM?**

Given the scope of this protocol, the details of agent-based modelling methods, variable selection and spatial analysis will be included in further papers.   In relation to the spatial analysis, we will use a Bernoulli geostatistical model to explore eventual spatial clustering of the prevalence of ESBL colonisation and determine which risk factors should be included in the ABM. The details of this are currently available online within the thesis of M Sammaro ( https://eprints.lancs.ac.uk/id/eprint/183677/). In relation to the ABM algorithms, these have been described online at https://arxiv.org/abs/2208.11331. We have therefore included these references in the text of the manuscript, amended the text to remove the “as described above” comment, and not expanded this section.

- **As this manuscript is a study protocol and investigating “the transmission of ESBL-E and ESBL-K between humans, animals, and the environment” was one of the study objectives, it would be valuable to add a section on genomic epidemiological methods to investigate the transmission (although, this may be further detailed in publication of the study results).**

This will indeed be further detailed in publication of the study results.

# Reviewer report: Reviewer response for version 1

**DOI:** 10.21956/wellcomeopenres.19440.r52055

**Published:** 2022-09-20

**Authors:** Catrin E Moore

**Affiliations:** 1 St George's, University of London, London, UK

Overview:

 The protocol describes an interdisciplinary study using a One Health approach which will characterise *E. coli *and *K. pneumoniae *strains carrying the extended spectrum beta lactamase resistance in the environment, animals and being carried by people in 400 households in Uganda and Malawi. The study provides a baseline examining the consequences of human behaviour, WASH practices, environmental contamination, and antibiotic use using longitudinal clinical and molecular microbiological data, set in urban and rural locations in both countries, before the introduction of relevant interventions. The study aimed to identify risk factors for and infer drivers of ESBL- *E. coli* and ESBL- *K. pneumoniae* transmission in Uganda and Malawi at the household level.

 The protocol is well written and all encompassing, describing a very holistic study to examine the drivers of antibiotic resistance in two low- and middle-income countries. I look forward to reading the results which will be published soon.

 I have some specific questions, separated by sections in the protocol:

Introduction:

- Please explain what you mean or provide a reference when you say “The 3rd-generation cephalosporin (3GC) ceftriaxone is frequently the antimicrobial agent of first and last resort across much of sSA”.
- You state that low-income countries in Africa have poor access to watch and reserve antibiotics, either provide a reference for this statement or explain this further with an additional sentence.
- What is a high incidence of sepsis and malaria? Please quantify.

Site descriptions:

- The site descriptions differ markedly between Malawi and Uganda, I would like to see a similar breakdown of the sites in Uganda as described in Malawi – the number of schools, farms, places of worship, hospitals and pharmacies etc.

Some clarification is required on the data collection questions on the Case Report Forms:

 01: Please write CHAM (facility) in full the first time.

 02: Please write CPPC in full the first time.

       If a patient received more than one antibiotic, how was the formulation (oral, syrup etc) recorded?

 03: Please add handwashing facilities (HWF) first time in the document.

 13: How did you determine the cloudiness of the water colour to ensure robustness?

Is the study design appropriate for the research question?

Yes

Is the rationale for, and objectives of, the study clearly described?

Yes

Are sufficient details of the methods provided to allow replication by others?

Yes

Are the datasets clearly presented in a useable and accessible format?

Not applicable

Reviewer Expertise:

microbiology, AMR, burden of AMR, antimicrobial use.

I confirm that I have read this submission and believe that I have an appropriate level of expertise to confirm that it is of an acceptable scientific standard.

# Response

**Published:** 2023-04-17

**Authors:** Derek Cocker

**Affiliations:** Liverpool School of Tropical Medicine, UK

Reviewer 1: **I have some specific questions, separated by sections in the protocol**

**Introduction:**

- **Please explain what you mean or provide a reference when you say “The 3rd-generation cephalosporin (3GC) ceftriaxone is frequently the antimicrobial agent of first and last resort across much of sSA”.**

Thank you for this comment. We mean that this is typically the agent used for empirical antimicrobial chemotherapy for individuals presented to hospital with features of severe infection, however if an isolate cultured from a clinical sample is resistant, there are typically no available agents that have broader spectrum (i.e. carbapenems). We have reworded the statement to read that *“* *The 3 ^rd^-generation cephalosporin (3GC) ceftriaxone is frequently the antimicrobial agent of choice for the empirical management of sepsis across much of sSA”* and referenced a paper on its use in one of the study states (Malawi) alongside global estimates. Reference additions (Lester et al. Clin Infect Dis. 2020 Dec 3;71(9):e478–86 & Browne AJ et al. Lancet Planet Health. 2021 Dec;5(12):e893-e904)

- **You state that low-income countries in Africa have poor access to watch and reserve antibiotics, either provide a reference for this statement or explain this further with an additional sentence.**

We have now cited in the manuscript recent data from Malawi, highlighting the precarity of antibiotic availability in rural settings, alongside global modelling data evidencing a lack of access. Reference additions (MacPherson EE et al. PLOS Glob Public Health. 2022 Jun;2(6):e0000314 and Browne AJ et al. Lancet Planet Health. 2021 Dec;5(12):e893-e904.)

- **What is a high incidence of sepsis and malaria? Please quantify.**

We have amended the manuscript to state that there is a “high burden of disease from neonatal sepsis and malaria”. The details of this are included in the newly added references attached for sepsis (Lewis et al, Crit Care. 2019 Jun 11;23(1):212, Rudd KE et al. Lancet. 2020 Jan 18;395(10219):200-211 & *Seale AC et al. Lancet Infect Dis. 2009 Jul;9(7):428–38*), and malaria (WHO. World Malaria report. Geneva, World Health Organization, Global Malaria Programme; 2021.). 

**Site descriptions:**

- **The site descriptions differ markedly between Malawi and Uganda, I would like to see a similar breakdown of the sites in Uganda as described in Malawi – the number of schools, farms, places of worship, hospitals and pharmacies etc.**

We regret that this level of detail could not be provided by the Uganda team prior to the study, as Uganda was more severely affected by pandemic lockdowns and the team was unable to generate these data. The study has no capacity to generate these data now, and the situation is likely to have changed. 

**Some clarification is required on the data collection questions on the Case Report Forms:**

**01: Please write CHAM (facility) in full the first time.** This has now been amended, and new versions of the CRFs are available online (https://doi.org/10.5281/zenodo.7836247). **02: Please write CPPC in full the first time. If a patient received more than one antibiotic, how was the formulation (oral, syrup etc) recorded?** This has now been amended, and new versions of the CRFs are available online (https://doi.org/10.5281/zenodo.7836247). For patients who received more than one antibiotic a separate entry was made for each antibiotic, inclusive of the formulation. This has now been made clearer on version 2 of the CRF, where is reminds the data collector to complete an entry for each antibiotic separately. **03: Please add handwashing facilities (HWF) first time in the document. ** This has now been amended, and new versions of the CRFs are available online (https://doi.org/10.5281/zenodo.7836247). **13: How did you determine the cloudiness of the water colour to ensure robustness?** Study staff will be trained using examples of where turbidity is considered clear, cloudy (mild), cloudy (moderate), cloudy (severe) for comparison. This assessment will then be made by the by study team at sample collection  by visual inspection, and not measured formally by nephelometric turbidity units (NTU), therefore, these parameters are subjective. River water colour is determined in a similar way.
